# Economic Feasibility of a New Method to Estimate Mortality in Crisis-Affected and Resource-Poor Settings

**DOI:** 10.1371/journal.pone.0025175

**Published:** 2011-09-19

**Authors:** Bayard Roberts, Oliver W. Morgan, Mohammed Ghaus Sultani, Peter Nyasulu, Sunday Rwebangila, Egbert Sondorp, Daniel Chandramohan, Francesco Checchi

**Affiliations:** 1 Faculty of Public Health and Policy, London School of Hygiene and Tropical Medicine, London, United Kingdom; 2 United States Centers for Disease Control and Prevention, Atlanta, Georgia, United States of America; 3 Médecins Sans Frontières France, Malawi Programme, Chiradzulu, Malawi; 4 United Nations High Commissioner for Refugees, Tanzania Office, Kigoma, Tanzania; 5 Faculty of Infectious and Tropical Diseases, London School of Hygiene and Tropical Medicine, London, United Kingdom; Ludwig Maximilian University of Munich, Germany

## Abstract

**Introduction:**

Mortality data provide essential evidence on the health status of populations in crisis-affected and resource-poor settings and to guide and assess relief operations. Retrospective surveys are commonly used to collect mortality data in such populations, but require substantial resources and have important methodological limitations. We evaluated the feasibility of an alternative method for rapidly quantifying mortality (the informant method). The study objective was to assess the economic feasibility of the informant method.

**Methods:**

The informant method captures deaths through an exhaustive search for all deaths occurring in a population over a defined and recent recall period, using key community informants and next-of-kin of decedents. Between July and October 2008, we implemented and evaluated the informant method in: Kabul, Afghanistan; Mae La camp for Karen refugees, Thai-Burma border; Chiradzulu District, Malawi; and Lugufu and Mtabila refugee camps, Tanzania. We documented the time and cost inputs for the informant method in each site, and compared these with projections for hypothetical retrospective mortality surveys implemented in the same site with a 6 month recall period and with a 30 day recall period.

**Findings:**

The informant method was estimated to require an average of 29% less time inputs and 33% less monetary inputs across all four study sites when compared with retrospective surveys with a 6 month recall period, and 88% less time inputs and 86% less monetary inputs when compared with retrospective surveys with a 1 month recall period. Verbal autopsy questionnaires were feasible and efficient, constituting only 4% of total person-time for the informant method's implementation in Chiradzulu District.

**Conclusions:**

The informant method requires fewer resources and incurs less respondent burden. The method's generally impressive feasibility and the near real-time mortality data it provides warrant further work to develop the method given the importance of mortality measurement in such settings.

## Introduction

Mortality data provide essential evidence on the health status of populations in crisis-affected and resource-poor settings and can help to guide and assess relief operations [1], [2]. Relief agencies most commonly measure mortality through retrospective surveys, in which a representative sample of households is interviewed using a standardised questionnaire about demographic changes (births, deaths, in- and out-migrations) in the household over a specified period in the past (the recall period). However, these surveys have serious feasibility limitations. First, they require significant time and resources to carry out because of the large sample sizes required (usually >900 households): this includes transport, hiring, training and supervising a team of interviewers, and entering data from a large number of questionnaires. Second, most data collection time during retrospective surveys is spent collecting data on living people (death is a comparatively rare event), which leaves less time for in-depth and reliable investigation of causes and circumstances of death. Third, retrospective surveys are poorly suited to investigating very recent periods (e.g. the last 1–2 months), as this entails interviewing a very large number of households (for example, estimating a crude mortality rate of 1 death per 10 000 per day with precision ±0.3 deaths per 10 000 per day over 1 month through a conventional two-stage cluster sample survey with a design effect of 2 and a mean household size of 5 would require a sample of 5691 households) [3], [4], [5], [6]. As a result, mortality surveys conducted in the humanitarian sector are not done frequently enough and often do not provide information useful for assessing programme performance. They also usually generate limited or unreliable data on causes of death, thereby reducing their usefulness for operational purposes [4], [7].

In a separate paper [8], we reported the validity of an alternative method for measuring mortality (the informant method) designed to provide estimates of mortality over a short recall period (i.e. almost on a real-time basis), and enable ascertainment of causes of death. We showed that the method's sensitivity, measured in four separate field settings against a best estimate of mortality derived through capture-recapture analysis, was moderate (55–73%), but encouraging enough to warrant further development. Elsewhere, we also reported findings of verbal autopsy questionnaires performed as part of the informant method in Malawi [9]. Here we report on the informant method's feasibility. The study objective was to assess the economic feasibility of the informant method.

## Methods

### Ethical approvals

Approval for this study was provided by the Ethics Committee of the London School of Hygiene and Tropical Medicine and all the national research ethical review boards where required. These were the Institutional Review Board of the Ministry of Public Health of Afghanistan; the National Health Sciences Research Committee in Malawi; and the National Institute for Medical Research and also the Commission for Science and Technology in Tanzania. In Thailand, no local institutional review board has jurisdiction over the refugee camps. Informed consent was received from all study participants. This consisted of information being provided to participants verbally and also through an information sheet in the local language which was given to the participants. Verbal consent from participants was sought and provided because there was limited literacy in all the study sites and so written consent was deemed inappropriate. Training on informed consent procedures and supervision of interviews was conducted to ensure informed consent procedures were adhered to. This approach was approved by the ethical review boards noted above.

### Study sites

Between July and October 2008, we evaluated the informant method in four study sites: District (*nahia*) 1 of Kabul, Afghanistan, a poor urban community; Mae La camp for Karen refugees, on the Thai-Burma border; Chiradzulu District in Malawi, a rural, scattered community impacted by the HIV pandemic; and Lugufu and Mtabila refugee camps (considered as one site for the purpose of this study) in Tanzania, which hosted refugees mainly from the Democratic Republic of Congo and Burundi. In Chiradzulu District, we implemented the informant method in a spatially representative sample of 96/757 villages (see Roberts et al [8] for detailed methods). Elsewhere, we implemented the method in the entire community.

### Implementation of the informant method

The informant method consists of an exhaustive search for all deaths occurring in a given community over a defined recall period, coupled with data on population size (denominator for the mortality rate) obtained either from available registration systems or by rapid estimation: thus allowing estimation of recent period mortality. The search process is mainly dependent on key community informants, selected through focus group discussions (FGDs), leading data collectors to households that they recalled as having experienced a recent death. Next-of-kin of decedents also act as key-informants to lead data collectors to other recently bereaved households. The process continues until informants can no longer identify households with recent deaths.

The method entails the following activities: (i) preparation, including seeking collaboration for the study from local authorities, translating and adapting data collection instruments into local languages, hiring data collectors and procuring supplies and logistics; (ii) training the data collectors; (iii) holding one FGD per study site, principally to identify key community informants for the exhaustive search for recent deaths, and featuring local participants with a presumed strong knowledge of how mortality information is shared in their community (community leaders, religious leaders, health workers, teachers, graveyard officials, etc.); (iv) carrying out the exhaustive search; (v) estimating population size, if a sufficiently reliable figure is not already available; (vi) entering and analysing data; and (vii) writing a report.

We conducted the exhaustive search for deaths by administrative sector (24 *guzars* in Kabul; 22 sections in Mae La camp; 96 villages in Chiradzulu; and 23 zones in Lugufu and Mtabila). In each sector, the key community informants identified through the FGDs (in Kabul, the *guzar* chief or *wakil* and *mullahs* of any mosque in the *guzar*; in Mae La, section leaders and section representatives of the Karen Women's Organisation; in Chiradzulu, village headmen and elder women or *fumukazi*; in Tanzania, zone leaders) referred the study team to any households in which they believed a death had occurred within the previous 60 days. A short, structured questionnaire was administered to consenting next of kin aged 18 years or older in which the date of death, age and sex of the decedent, and the cause and place of death were recorded. Respondents were also asked to recall other deaths in their household or in their community within the previous 60 days. The sector was considered exhausted when neither key informants nor households informants could identify additional bereaved households.

In Mae La and the Tanzanian camps, we used existing data on population size from well-established prospective demographic surveillance systems. Elsewhere, population data were unreliable and we did our own estimation of population size. In Kabul we divided District 1 into a high- and low-lying stratum: in the former, we estimated population size using a combination of residential structure counts and a small structure occupancy survey; in the latter, we divided the total area into quadrants, and estimated population density based on a sample of quadrants with the use of a high resolution map of the District. In Chiradzulu district, it was considered inefficient to visit all 757 villages. Instead, we surveyed a fraction, selected using a modified centric systematic area sampling design. We divided the district into 32 five Km × five Km quadrats with area mostly falling within the district boundaries. Using high-resolution maps, we then selected the three villages closest to the centre of each quadrat, thus yielding a non-self-weighting sample of 96 villages. To estimate population size in Chiradzulu, we counted residential structures in each village and estimated their average occupancy using a small nested survey. For a more detailed description of these methods, please see Roberts et al [8].

### Feasibility evaluation

During the field testing, we systematically recorded time inputs in hours and minutes for paid staff, study participants, and community informants on standardised forms. We also systematically recorded the cost in local currency of activities by staff type. These included salary payments and associated costs such as for vehicle hire and fuel which were included under the driver staff type. Costs for key informants, FGD participants, and respondents were not included as they did not receive any payment. The activities and staff types included in the analysis are shown in the first column of Table 1. We computed the total person-hours and financial cost for implementation of the informant method, as well as the time and cost required to detect each death. The local currency costs were converted to US dollars at local exchange rates on the first data collection day in each study site (see Table 2 for exchange rates used).

**Table 1:** Activities, staff types, and assumptions for time inputs for a retrospective survey, by site.

Activity/Staff type	Assumptions for time inputs for a retrospective survey			
	District 1, Kabul	Mae La camp	Chiradzulu District	Tanzania camps
**Preparation:**				
Investigators	same as for informant method	same as for informant method	same as for informant method	same as for informant method
Data collectors				
Collaborators				
Drivers				
**Group discussion:**	not applicable for survey	not applicable for survey	not applicable for survey	not applicable for survey
**Training:**				
Investigators	1 investigator × 4 days	1 investigator × 4 days	1 investigator × 4 days	1 investigator × 4 days
Data collectors	12 interviewers (teams of 2†) × 4 days (recall 6 months)	6 interviewers × 4 days (recall 6 months)	6 interviewers × 4 days (recall 6 months)	6 interviewers × 4 days (recall 6 months)
	80 interviewers (teams of 2) × 4 days† (recall 30 days)	40 interviewers × 4 days (recall 30 days)	40 interviewers × 4 days (recall 30 days)	40 interviewers × 4 days (recall 30 days)
**Data collection:**				
Investigators	person-time for data collectors/n of data collectors (n = 12 for 6 month recall period; 80 for 3 0day recall period)	person-time for data collectors/n of data collectors (n = 6 for 6 month recall period; 40 for 30 day recall period)	person-time for data collectors/n of data collectors (n = 6 for 6 month recall period; 40 for 30 day recall period)	person-time for data collectors/n of data collectors (n = 6 for 6 month recall period; 40 for 30 day recall period)
Data collectors	1 h preparation/cluster	15 min/household	1 h preparation/cluster	15 min/household
			1 h drive/cluster	3 min to select each new household
	15 min/household	3 min to select each new household	15 min/household questionnaire	1 data collector/household
	3 min to select each new household	1 data collector/household	3 min to select each new household	
	2 data collectors/household		1 data collector/household	
Key informants*	30 min × 1 informant/cluster	30 min × 22 section chiefs	30 min × 1 informant/cluster	30 min × 35 section chiefs
Drivers	2 drivers × person-time for study investigators	No driving necessary	2 drivers × person-time for study investigators	1 driver × person-time for study investigator
Respondents	1 person/household × 15 min	1 person/household × 15 min	1 person/household × 15 min	1 person/household × 15 min
**Data entry/analysis:**				
Investigators	entry: 3 min/questionnaire + 20% double entry	entry: 3 min/questionnaire + 20% double entry	entry: 3 min/questionnaire + 20% double entry	entry: 3 min/questionnaire + 20% double entry
	analysis: 1 investigator × 2 days	analysis: 1 investigator × 2 days	analysis: 1 investigator × 2 days	analysis: 1 investigator × 2 days
**Report production:**				
Investigators	1 investigator × 2 days	1 investigator × 2 days	1 investigator × 2 days	1 investigator × 2 days

Abbreviations: n, number; h, hour; min, minutes.

† Kabul has double the investigators of other sites as 1 woman and 1 man were needed for interviews.

*Key informants used help ensure community support and to and guide the team around the community.

Note: Working day assumed to be 8 hours.

**Table 2:** Costings for staff used for the informant method and a retrospective mortality survey, by site.

Staff	Hourly rate (US$)			
	District 1, Kabul	Mae La Camp	Chiradzulu District	Tanzania camps
Study investigators	22.6	22.6	22.6	22.6
Other staff†	5.3	4.5	264*	3.3
Collaborators	5.4	9.9	5.7	3.5
Data collectors	3.9	4.5	2.3	3.1
Drivers¥	4.7		27	2.2

† Other staff include people used for one off activities such as population estimation.

*In Chiradzulu a one-off payment was made to other study staff (household enumerators for population data) amounting to $264 in total.

¥ Includes costs of vehicles hire, driver fees, petrol.

Notes:

Hourly staff costs are based on those incurred during implementation of informant method.

Costs for key informants, FGD participants, and respondents are not included as payments are not usually made to these individuals.

The average cost for different study staff in each study site is reported.

Exchange rates are as recorded on the first data collection day in each study site: 1 USD = 50.20 Afghani (14 July 2008); 1 USD = 33.87 Thai Baht (11 July 2008); 1 USD = 143.29 Malawi Kwacha (26 August 2008; 1 USD = 1186.53 Tanzanian shillings (3 October 2008).

We then compared the observed time and cost inputs for implementing the informant method in each study site with those estimated for a conventional retrospective mortality survey. To do this, we assumed that a retrospective survey would be conducted in each of the four sites broadly in line with guidelines described in the Standardised Monitoring and Assessment in Relief and Transition (SMART) initiative (www.smartindicators.org) [10]. Taking into account the layout and organisation of the four sites, we assumed that cluster sampling with probability proportional to size (PPS) would be used in Kabul and Chiradzulu, and linear systematic random sampling in the Mae La and Tanzania camps, where household lists were available. We assumed that a survey with 6 months recall would feature typical sample sizes of 30 clusters of 32 households in Kabul and Chiradzulu (i.e. 960 households in total, based on a design effect of 2.0), and 480 households (i.e. the same effective sample size, but with no design effect) in Mae La and Tanzania. The above sample sizes are adequate to detect a crude mortality rate over 6 months of 2, 1 or 0.5 deaths per 10 000 person-days with an absolute precision of ±0.4, 0.3 and 0.2 deaths per 10 000 person-days respectively (i.e. a relative precision no worse than ±40%), assuming a mean household size of five persons. We also compared the informant method to a survey with a 30 day recall (i.e. the same as the informant method), for which we assumed a sample size of 200 clusters of 32 households (6402 households; cluster sampling with design effect = 2.0) or 3201 households (no design effect), needed to obtain relative precision no worse than ±40% if the crude mortality rate is ≥0.5 per 10 000 person-days. Other assumptions for time inputs for a retrospective survey are reported in Table 1.

Estimated economic costs were multiplied by time inputs for both the informant method and retrospective mortality surveys to enable an economic cost-based comparison between the two methods. The costs for a hypothetical retrospective survey were calculated for each site using the unit costs recorded for implementing the informant method (Table 2).

We also evaluated the feasibility of adding a verbal autopsy (VA) component to the informant method in the Chiradzulu District site. This was evaluated only in one site due to budget constraints of the research project. We also felt that one site would provide sufficient evidence for the use of verbal autopsy with the informant method. We selected Chiradzulu district because the partner agency (Médecins Sans Frontières-France) expressed a desire to measure cause-specific mortality and had the capacity to use such data for its operational interventions. We used the most recent, standardised World Health Organization VA questionnaires (http://www.who.int/whosis/mort/verbalautopsystandards/en/index.html) [11], [12]. These consist of three different questionnaires according to the age of the decedent (less than four weeks; four weeks to 14 years; older than 14 years), and containing several sub-modules (e.g. on neonatal conditions, injuries, and maternal mortality) depending on the signs and symptoms reported by the respondent. The questionnaires were translated into Chichewa by a clinical officer, and translations were reviewed by another clinician as well as other members of the study team. If during the exhaustive search, a death was established as taking place within a 30 day recall period, the respondent was asked to also participate in a separate VA interview, conducted by a clinical officer. We timed each verbal autopsy interview, as well as time required for its analysis according to World Health Organization guidelines (parallel review by two independent clinicians, and an additional review by a third clinician in case of discrepant classification of cause of death by the first two). Details on causes of death as ascertained through VA in Chiradzulu are reported elsewhere [9].

## Results

Descriptive information on the population size, data collection period, response rate, mortality data collected by the informant method and its sensitivity is given in Table 3. All key community informants who were found agreed to provide information. In Chiradzulu District, two households delayed their consent after consulting with family members or the headman. In Tanzania, one household refused to give consent.

**Table 3:** Timeframe, population covered, response rate, mortality data and sensitivity recorded by the informant method, by site (from Roberts et al. [8]).

Parameter	District 1, Kabul	Mae La camp	Chiradzulu District	Tanzania camps
Population size (age<5 years)	76 476 (13 790)	43 794 (5 384)	54 418 (9 462)	80 136 (16 028)
Data collection timeframe	14–27 July 2008	11–17 July 2008†	26 August–16 September 2008	3–9 October 2008
Household response rate	100%	100%	100%	98%
Deaths (age<5 years)*	67(20)	27(2)	93(26)	44(22)
CMR (95%CI)*	0.15 (0.12–0.19)	0.10 (0.09–0.11)	0.30 (0.23–0.39)	0.09 (0.09–0.10)
U5MR (95%CI)*	0.24 (0.17–0.33)	0.06 (0.06–0.07)	0.54 (0.30–0.93)	0.23 (0.21–0.24)
Sensitivity (%) over a 60 days recall period (95%CI)¥	62.6 (39.9–72.8)	45.0 (37.0–48.2)	65.0 (47.9–75.6)	53.0 (36.4–62.9)
Sensitivity (%) over a 30 days recall period (95%CI)¥	55.0 (37.9–61.1)	64.0 (50.0–69.6)	72.5 (46.8–82.2)	67.7 (51.2–72.4)

† Two interviews were conducted on 27 July due to previous inability to contact the household.

*As recorded by the informant method (60 day recall period).

¥ The sensitivity of the informant method was estimated by comparing the number of deaths recorded by the informant method with a best estimate of mortality using capture-recapture analysis (for full results see Roberts et al. [8]).

Abbreviations: CI, confidence interval; CMR, crude mortality rate; U5MR, under-five mortality rate.

Note: Detailed information on the informant method's mortality and validation results is provided elsewhere.

The summary time and cost inputs for conducting the informant method in each of the four sites are given in Table 4 (please see Table S1 for detailed activity time and cost inputs by staff type). There was significant variance in the time-inputs among the study sites. For example, the study in Mae La camp required only 168 person-hours (21 person-working days) to be completed compared to 2069 (259 person-working days) in Chiradzulu District.

**Table 4:** Summary time and cost inputs of the informant method and retrospective surveys with 6 month and 30 day recall periods, by site and activity.

Activity	Time inputs in person hours (cost inputs in US$)																							
	District 1, Kabul						Mae La Camp						Chiradzulu District						Tanzania camps					
	Informantmethod		Survey:6 month recall		Survey:30 day recall		Informantmethod		Survey:6 month recall		Survey:30 day recall		Informantmethod		Survey:6 month recall		Survey:30 day recall		Informantmethod		Survey:6 month recall		Survey:30 day recall	
Preparation	199	(2543)	199	(2543)	199	(2543)	16	(238)	16	(238)	16	(238)	185	(2668)	185	(2668)	185	(2668)	59	(663)	59	(663)	59	(663)
Population estimation§	107	(1076)	0	(0)	0	(0)	0	(0)	0	(0)	0	(0)	1003	(2595)	0	(0)	0	(0)	0	(0)	0	(0)	0	(0)
FGD	179	(1279)	0	(0)	0	(0)	88	(257)	0	(0)	0	(0)	67	(364)	0	(0)	0	(0)	118	(210)	(0)	(0)	0	(0)
Training	171	(1165)	416	(2202)	2593	(10584)	6	(63)	224	(1587)	1313	(6486)	160	(957)	224	(1165)	1313	(3669)	71	(571)	224	(1318)	1313	(4693)
Data collection	810	(5513)	1425	(6101)	9420	(40690)	53	(393)	299	(1190)	1994	(7940)	625	(7919)	1227	(9756)	8184	(65076)	177	(699)	330	(1042)	2099	(6947)
Data entry/analysis	8	(181)	82	(1853)	400	(9044)	1	(23)	45	(1017)	208	(4703)	21	(475)	82	(1853)	400	(9044)	9	(203)	45	(1017)	208	(4703)
Report production	8	(181)	16	(362)	16	(362)	4	(90)	16	(362)	16	(362)	8	(181)	16	(362)	16	(362)	10	(226)	16	(362)	16	(362)
**Total time (costs)**	**1481**	**(11933)**	**2138**	**(13060)**	**12628**	**(63223)**	**168**	**(1065)**	**600**	**(4395)**	**3547**	**(19729)**	**2069**	**(15158)**	**1734**	**(15804)**	**10098**	**(80818)**	**444**	**(2572)**	**674**	**(4401)**	**3695**	**(17368)**

See text and Tables 1 and 2 for assumptions for time and cost inputs respectively for a retrospective surveys.

See Table S1 for detailed time and cost inputs by staff type.

§ Population estimation only included if required in study.

When compared to a survey with a 6 month recall period, the informant method was estimated to require 658 (31%) fewer person-hours in Kabul, 432 (72%) in Mae La camp, and 230 (34%) in the Tanzania camps than the survey. However, in Chiradzulu District, the informant method was estimated to require 335 (19%) more person-hours than the survey (Table 4). If only study staff time is considered (i.e. excluding respondents, participants and key-informants), the informant method was estimated to require 509 (27%) fewer person-hours in Kabul, and 390 (83%) fewer person-hours in Mae La camp, and 238 (44%) fewer person-hours in the Tanzania camps, but 438 (30%) more person-hours in Chiradzulu District. Population estimation accounted for only 107/1482 (7%) person-hours of the informant method in Kabul, but considerably more (1003/2069 or 48%) in Chiradzulu (Table S1 and Table 5).

**Table 5:** Time and cost inputs of the informant method and retrospective surveys with 6 month and 30 day recall periods, by site and staff type.

Staff type	Time inputs in person-hours (cost inputs in US$)																							
	Afghanistan						Thailand						Malawi						Tanzania					
	Informantmethod		Survey(6 month recall)		Survey(30 day recall)		Informantmethod		Survey(6 month recall)		Survey(30 day recall)		Informantmethod		Survey(6 month recall)		Survey(30 day recall)		Informantmethod		Survey(6 month recall)		Survey(30 day recall)	
Investigators*	336	(7582)	298	(6735)	1058	(23921)	38	(859)	125	(2825)	424	(9586)	258	(5831)	283	(6396)	1214	(27426)	85	(1921)	141	(3187)	440	(9948)
Other staff	104	(546)	0	(0)	0	(0)	14	(63)	0	(0)	0	(0)	849	(264)	0	(0)	0	(0)	18	(59)	0	(0)	0	(0)
Data collectors	721	(2774)	1345	(5178)	8829	(33993)	23	(104)	340	(1530)	2245	(10103)	494	(1136)	902	(2075)	5665	(13029)	131	(406)	342	(1060)	2247	(6966)
Drivers	184	(864)	215	(1012)	1100	(5174)	0	(0)	0	(0)	0	(0)	288	(7767)	266	(7174)	1491	(40203)	30	(66)	24	(53)	160	(352)
Collaborators	31	(167)	25	(135)	25	(135)	4	(40)	4	(40)	4	(40)	28	(160)	28	(160)	28	(160)	34	(119)	29	(102)	29	(102)
Respondents§ **	24	(0)	240	(0)	1601	(0)	8	(0)	120	(0)	800	(0)	25	(0)	240	(0)	1601	(0)	16	(0)	120	(0)	800	(0)
FGD participants**	24	(0)	0	(0)	0	(0)	67	(0)	0	(0)	0	(0)	33	(0)	0	(0)	0	(0)	83	(0)	0	(0)	0	(0)
Key-informants**	58	(0)	15	(0)	15	(0)	14	(0)	11	(0)	73	(0)	94	(0)	15	(0)	100	(0)	47	(0)	18	(0)	18	(0)
***Total time (costs)***	**1481**	**(11933)**	**2138**	**(13060)**	**12628**	**(63223)**	**168**	**(1065)**	**600**	**(4395)**	**3547**	**(19729)**	**2069**	**(15158)**	**1734**	**(15804)**	**10098**	**(80818)**	**444**	**(2572)**	**674**	**(4401)**	**3695**	**(17368)**

See text and Tables 1 and 2 for assumptions for time and cost inputs respectively for retrospective surveys.

§ Respondents for data collection and population estimation.

*Investigator time inputs are for implementing the informant method, rather than evaluating its validity and feasibility.

**Costs not attached as they are not normally paid to respondents, participants and key-informants.

The total cost of the informant method ranged from $1065 in Mae La camp to $15158 in Chiradzulu. The informant method was estimated to cost $1127 (9%) less than a survey with 6 months recall period in Kabul, $3330 (76%) less in Mae La camp, $646 (4%) less in Chiradzulu, and $1830 (42%) less in Tanzania (Table 4).

When the informant method is compared to a survey with a 30 day recall period, the informant method was estimated to require 11148 (88%) fewer person-hours in Kabul, 3379 (95%) fewer in Mae La camp, 8029 (80%) fewer in Chiradzulu, and 3251 (88%) fewer in the Tanzania camps. The informant method also cost $51290 (81%) less than a survey in Kabul, $18664 (95%) less in Mae La camp, $65660 (81%) less in Chiradzulu, and $14796 (85%) less in Tanzania.

Table 5 presents time and cost inputs by staff type. The informant method required substantially less time inputs for data collectors and particularly respondents in all four study sites when compared with retrospective surveys. Less time was spent by study investigators on the informant method compared with retrospective surveys, with the exception of Kabul (when compared with a 6 month recall survey): here two investigators were present as this was the first site in which the informant method was tested. The ‘other staff’ category recorded more time input for the informant method than a survey. This was particularly the case in Chiradzulu District due to the time input by village residents hired to perform structure counts for the population estimation. The informant method incurred a lower burden to respondents than a retrospective survey in terms of respondent time inputs: 216 (90%) fewer respondent hours in Kabul; 112 (93%) fewer in Mae La camp, 215 (90%) fewer in Chiradzulu and 104 (87%) fewer in the Tanzania camps. These differences are even greater when compared with 30 day recall survey, with over 98% fewer respondent hours in all the study sites.

The time (and US$ cost) required per death detected using the informant method was 5 hours ($28) in the Tanzania camps, 6 hours ($39) in Mae La camp, 22 hours ($178) in District One of Kabul, and 23 hours ($163) in Chiradzulu District.

For the feasibility of adding verbal autopsy questionnaires to the informant method, each interview for the verbal autopsy questionnaire took an average of 38 minutes, and 56 verbal autopsy questionnaires were completed in Chiradzulu District. Each questionnaire took an average of 44 minutes to analyse (including time input by all questionnaire reviewers). The total time to conduct the verbal autopsy component was therefore 76.5 person-hours (4% of total person-time for the informant method's implementation in Chiradzulu District).

## Discussion

This study presents findings on the economic feasibility of a new method to estimate mortality in crisis-affected and resource-poor settings, based on field evaluations in four different sites.

There was significant variance in the time-inputs among the study sites, reflecting their different characteristics. The informant method in Mae La camp in Thailand and the Tanzania camps required only 168 person-hours and 444 person-hours respectively. This was because little travel and data collection time were required due to the low number of deaths recorded and high population density of these camp settings. They also did not require population estimation to be conducted. The study in Tanzania required greater time inputs than Mae La camp principally because two camps were included. By contrast, the study in Chiradzulu District recorded 93 deaths in 96 villages covering an area of approximately 875 km^2^, and required population estimation, which accounted for almost half of total person-hours (most of which was attributable to villagers hired to count structures). The Kabul site also entailed high person-time inputs, partly due to the requirement to hire additional data collection staff to ensure same-sex interviews and FGDs; population estimation in Kabul, on the other hand, accounted for a small proportion of total person-time (7% of total person hours).

Comparisons between the informant method and conventional retrospective surveys suggest that the informant method could offer considerable economic benefits over retrospective surveys. The informant method required less time than retrospective surveys with a 6 month recall period in three of the four study sites. It also showed considerable monetary savings, with staff cost estimates in all four study sites lower for the informant method than for a survey. In future studies, where accurate population data (for all ages and for under 5 years) are not available, population estimation would be required, stratifying the estimate by age group, as we did in this study for the Kabul and Chiradzulu District study sites. However, even where population estimation is required, the results from our study suggest that the informant method would require less time and monetary inputs than for a survey. Importantly, the recall period for the informant method was 1 month which would be of much greater operational use for relief agencies than a survey with a 6 month recall period, particularly in acute emergencies in which humanitarian agencies need recent mortality data. When the results of the informant method were compared against a retrospective survey with the recall period of 1 month, the time and costs benefits of the informant method increased substantially. However, a drawback of the informant method is that it only measures mortality, whereas retrospective surveys can include other outcomes, such as child nutrition and micronutrient data and vaccination status at little extra cost [10].

Data entry is substantially less for the informant method than retrospective surveys. If the entire community is surveyed exhaustively, data analysis is also simpler than for surveys by removing the need for weighting and design effect adjustment inherent in sample surveys, or individual person-time calculation when the recall period is long and the cohort very dynamic. The method could therefore be used by programme staff with limited research skills.

Generally, we believe that the informant method would be most feasible in camps and concentrated populations. In post-emergency camps or other communities where population size is being monitored, feasibility results from Mae La and the Tanzania camps represent what might be routinely expected. In chaotic situations where no population estimates are available, time and costs of the population size estimation required for the informant method would be expected to increase.

A major benefit of the informant method was that respondent burden was considerably less than for retrospective mortality surveys, with approximately 90% less time spent by informants on the informant method compared with surveys with a 6 month recall period (rising to over 98% for surveys with a 30 day recall period). Surveys can be burdensome for communities, and reducing respondent time has ethical implications [13]. In addition, all key community informants who were found agreed to provide information, and household response was almost 100%.

However, a number of potential ethical issues regarding willingness to participate potentially exist. The referral by key informants who are also community leaders could infringe on the principle of voluntary participation. The informant method exploits hierarchical social structures: deference towards and/or fear of authorities may mean that households might be unable to refuse participation in the study, or decide which information about the decedent they wish to disclose (while introducing bias into estimation, deliberately false answers may be a justifiable way for the household to protect itself against the consequences of sharing certain information with strangers). We did not observe or hear of incidents suggesting any of these dynamics, although we recognise that such delicate issues are difficult to gauge in the short amount of time we spent in each study location. Moreover, the method's reliance on key informants or neighbours to identify households with deaths essentially deprives those households of the choice to disclose the death to investigators, and to some extent to disclose details on the decedent. We also found it difficult to persuade key informants not to be present during the interview (no key-informants were actually present during the interviews but they were present during the initial introductions for around 20% of the households visited), but they should be actively discouraged from being present during the interview to ensure full confidentiality. Follow-up work could be conducted to explore any untoward effects of the referral system that underpins the informant method, and find ways to strengthen the consent and confidentiality arrangements. However, it should also be recognised that similar ethical issues around voluntary participation exist in retrospective surveys which often seek permission for elders and village leaders and such permission can also place pressure on respondents to participate.

The addition of verbal autopsy questionnaires in the Chiradzulu District site took a total additional time of 76.5 hours, 4% of the total person-time input in that site. We believe this suggests that it is feasible to routinely add verbal autopsy questionnaires to the informant method given the reduced time required for conducting the informant method when compared to a retrospective survey. The addition of verbal autopsy questionnaires when measuring mortality rates provides an extremely important way of increasing the accuracy of recording the causes of mortality and so helping to inform appropriate health interventions and responses.

There was a very high willingness of households to respond. If response rates were lower then it would reduce the sensitivity and economic feasibility of the method. However, we do not feel that these high response rates were due to the particular research situations in our study, and the varied settings and populations used for the study provide a good indication that such response rates could be expected elsewhere. We also do not believe there were any aspects of our own research approach that meant response rates were higher in our study than if the informant method was used by other researchers applying sufficient levels of care and the same basic principles and standards required for research in such settings [10].

The informant method also had only moderate sensitivity and the next steps in achieving better sensitivity would include the use of ethnographic research on how information of death is shared in a community, in a variety of settings, to help refine the informant method procedures and questionnaires for future use. Another round of validating the method in a few more sites, focussing on increasing sensitivity through more effective use of key informants, would also be beneficial. Such sites should include unstable displaced camps with fluctuating populations.

### Study Limitations

The study has a number of limitations. First, we were unable to test the method in settings of an acute humanitarian crisis where mortality is likely to be highest and where we believe the method would be most useful. This was because we needed a sufficiently stable environment to conduct the validation aspect of this study (from Roberts et al. [8]). Second, we were unable to compare the time and costs with actual retrospective mortality surveys as none were conducted in directly comparable locations and time and recall periods. We therefore had to rely on estimations based on assumptions. The time-based approach excluded non-time-based parameters such as materials and supplies (e.g. food, communications, photocopies). However, these non-time-based items are likely to have contributed only a small proportion of costs and so would only have a minor influence on the overall costings. Lastly, the limited sensitivity of the informant method means costs could potentially increase in order to improve the referral information (e.g. use of more key informants) but we also believe that improving sensitivity principally involves more effective selection of key informants and better eliciting of information from them which would not necessarily require additional resources. Improving sensitivity would also mean interviewing more people which would take more time and money. Although it should be recognised that the actual data collection accounted for about 30–50% of total costs, and most of these costs related to driving to sites, making contact with key informants, and obtaining their list of recent deaths, with little incremental cost due to additional interviews. If sensitivity went up from the current 60–70% to 100%, data collection costs would certainly rise, but the overall effect on the total budget of the study would probably be less than 10%. However, our data was not sufficiently detailed to contain the incremental costs required to accurately estimate the additional costs resulting from increased sensitivity.

### Conclusions

The validation of the informant method indicated that further work is required to improve the informant method's moderate sensitivity which ranged from 55% to 73% and which was comparable with the well established surveillance systems in Mae La camp and the Tanzania camps, but below the sensitivity level of 80% that we had aimed for when developing the method (see Table 3, and Roberts et al. [8] for further details). However, the informant method requires fewer resources and incurs less respondent burden, and allows for more time to add additional investigation components such as verbal autopsy questionnaires to obtain more reliable information on the causes of death. We believe that the generally impressive feasibility of the informant method and the near real-time mortality data it provides warrant further work to develop the method, given the paramount importance of mortality measurement and the limitations of current methods to measure mortality.

## Supporting Information

Table S1 **Detailed time and cost inputs of the informant method and retrospective surveys with 6 month and 30 day recall periods, by site, activity and staff type.**
(DOC)
