# Supplementary material for: Economic Feasibility of a New Method to Estimate Mortality in Crisis-Affected and Resource-Poor Settings
Source: PLoS One. 2011 Sep 19;6(9):e25175. doi: 10.1371/journal.pone.0025175 (PMC3176324; doi:10.1371/journal.pone.0025175)
Supplement: Table S1 — Detailed time and cost inputs of the informant method and retrospective surveys with 6 month and 30 day recall periods, by site, activity and staff type. (DOC) [file pone.0025175.s001.doc]

Supporting Information

Table S1: Detailed time and cost inputs of the informant method and retrospective surveys with 6 month and 30 day recall periods, by site, activity and staff type.

| **Activity/**  Staff type | **Time inputs in person hours (cost inputs in US$)** | | | | | | | | | | | | | | | | | | | | | | | |
| --- | --- | --- | --- | --- | --- | --- | --- | --- | --- | --- | --- | --- | --- | --- | --- | --- | --- | --- | --- | --- | --- | --- | --- | --- |
|  | **District 1, Kabul** | | | | | | **Mae La Camp** | | | | | | **Chiradzulu District** | | | | | | **Tanzania camps** | | | | | |
|  | **Informant**  **method** | | **Survey:**  **6 month recall** | | **Survey:**  **30 day recall** | | **Informant**  **method** | | **Survey:**  **6 month recall** | | **Survey:**  **30 day recall** | | **Informant**  **method** | | **Survey:**  **6 month recall** | | **Survey:**  **30 day recall** | | **Informant**  **method** | | **Survey:**  **6 month recall** | | **Survey:**  **30 day recall** | |
| **Preparation** |  |  |  |  |  |  |  |  |  |  |  |  |  |  |  |  |  |  |  |  |  |  |  |  |
| Study investigators* | 90 | (2034) | 90 | (2034) | 90 | (2034) | 8 | (181) | 8 | (181) | 8 | (181) | 45 | (1017) | 45 | (1017) | 45 | (1017) | 24 | (542) | 24 | (542) | 24 | (542) |
| Data collectors | 25 | (96) | 25 | (96) | 25 | (96) | 4 | (18) | 4 | (18) | 4 | (18) | 62 | (143) | 62 | (143) | 62 | (143) | 6 | (19) | 6 | (19) | 6 | (19) |
| Drivers | 59 | (278) | 59 | (278) | 59 | (278) | 0 | (0) | 0 | (0) | 0 | (0) | 50 | (1348) | 50 | (1348) | 50 | (1348) | 0 | (0) | 0 | (0) | 0 | (0) |
| Collaborators | 25 | (135) | 25 | (135) | 25 | (135) | 4 | (40) | 4 | (40) | 4 | (40) | 28 | (160) | 28 | (160) | 28 | (160) | 29 | (102) | 29 | (102) | 29 | (102) |
| *Sub(total* | *199* | *(2543)* | *199* | *(2543)* | *199* | *(2543)* | *16* | *(238)* | *16* | *(238)* | *16* | *(238)* | *185* | *(2668)* | *185* | *(2668)* | *185* | *(2668)* | *59* | *(663)* | *59* | *(663)* | *59* | *(663)* |
| **Population estimation** § |  |  |  |  |  |  |  |  |  |  |  |  |  |  |  |  |  |  |  |  |  |  |  |  |
| Study investigators* | 36 | (802) | 0 | (0) | 0 | (0) | 0 | (0) | 0 | (0) | 0 | (0) | 27 | (610) | 0 | (0) | 0 | (0) | 0 | (0) | 0 | (0) | 0 | (0) |
| Other study staff | 6 | (32) | 0 | (0) | 0 | (0) | 0 | (0) | 0 | (0) | 0 | (0) | 849 | (264) | 0 | (0) | 0 | (0) | 0 | (0) | 0 | (0) | 0 | (0) |
| Data collectors | 63 | (243) | 0 | (0) | 0 | (0) | 0 | (0) | 0 | (0) | 0 | (0) | 68 | (156) | 0 | (0) | 0 | (0) | 0 | (0) | 0 | (0) | 0 | (0) |
| Respondents** | 2 | (0) | 0 | (0) | 0 | (0) | 0 | (0) | 0 | (0) | 0 | (0) | 1 | ( (0) | 0 | (0) | 0 | (0) | 0 | (0) | 0 | (0) | 0 | (0) |
| Drivers | 0 | (0 | 0 | (0) | 0 | (0) | 0 | (0) | 0 | (0) | 0 | (0) | 58 | (1564) | 0 | (0) | 0 | (0) | 0 | (0) | 0 | (0) | 0 | (0) |
| *Sub(total* | *107* | *(1076)* | *0* | *(0)* | *0* | *(0)* | *0* | *(0)* | *0* | *(0)* | *0* | *(0)* | *1003* | *(2595)* | *0* | *(0)* | *0* | *(0)* | *0* | *(0)* | *0* | *(0)* | *0* | *(0)* |
| **FGD** |  |  |  |  |  |  |  |  |  |  |  |  |  |  |  |  |  |  |  |  |  |  |  |  |
| Study investigators* | 34 | (768) | 0 | (0) | 0 | (0) | 9 | (203) | 0 | (0) | 0 | (0) | 8 | (181) | 0 | (0) | 0 | (0) | 5 | (113) | (0) | (0) | 0 | (0) |
| Other study staff | 22 | (116) | 0 | (0) | 0 | (0) | 9 | (41) | 0 | (0) | 0 | (0) |  | (0) | 0 | (0) | 0 | (0) | 10 | (33) | (0) | (0) | 0 | (0) |
| Participants** | 24 | (0) | 0 | (0) | 0 | (0) | 67 | (0) | 0 | (0) | 0 | (0) | 33 | (0) | 0 | (0) | 0 | (0) | 83 | (0) | (0) | (0) | 0 | (0) |
| Data collectors | 87 | (335) | 0 | (0) | 0 | (0) | 3 | (14 | 0 | (0) | 0 | (0) | 21 | (48) | 0 | (0) | 0 | (0) | 15 | (47) | (0) | (0) | 0 | (0) |
| Collaborators | 6 | (32) | 0 | (0) | 0 | (0) | 0 | (0) | 0 | (0) | 0 | (0) |  | (0) | 0 | (0) | 0 | (0) | 5 | (18) | (0) | (0) | 0 | (0) |
| Drivers | 6 | (28) | 0 | (0) | 0 | (0) | 0 | (0) | 0 | (0) | 0 | (0) | 5 | (135) | 0 | (0) | 0 | (0) | 0 | (0) | (0) | (0) | 0 | (0) |
| *Sub(total* | *179* | *(1279)* | *0* | *(0)* | *0* | *(0)* | *88* | *(257)* | *0* | *(0)* | *0* | *(0)* | *67* | *(364)* | *0* | *(0)* | *0* | *(0)* | *118* | *(210)* | *(0)* | *(0)* | *0* | *(0)* |
| **Training** |  |  |  |  |  |  |  |  |  |  |  |  |  |  |  |  |  |  |  |  |  |  |  |  |
| Study investigators* | 27 | (610) | 32 | (723) | 32 | (723) | 2 | (45) | 32 | (723) | 32 | (723) | 29 | (655) | 32 | (723) | 32 | (723) | 18 | (407) | 32 | (723) | 32 | (723) |
| Data collectors | 144 | (554) | 384 | (1478) | 2561 | (9861) | 2 | (9) | 192 | (864) | 1281 | (5763) | 131 | (301) | 192 | (442) | 1281 | (2945) | 53 | (164) | 192 | (595) | 1281 | (3970) |
| Other study staff | 0 | (0) | 0 | (0) | 0 | (0) | 2 | (9) | 0 | (0) | 0 | (0) | 0 | (0) | 0 | (0) | 0 | (0) ) | 0 | (0) | 0 | (0) | 0 | (0) |
| *Sub(total* | *171* | *(1165)* | *416* | *(2202)* | *2593* | *(10584)* | *6* | *(63)* | *224* | *(1587)* | *1313* | *(6486)* | *160* | *(957)* | *224* | *(1165)* | *1313* | *(3669)* | *71* | *(571)* | *224* | *(1318)* | *1313* | *(4693)* |
| **Data collection** |  |  |  |  |  |  |  |  |  |  |  |  |  |  |  |  |  |  |  |  |  |  |  |  |
| Study investigators* | 133 | (3006) | 78 | (1763) | 520 | (11758) | 14 | (316) | 24 | (542) | 160 | (3618) | 120 | (2712) | 108 | (2441) | 720 | (16280) | 19 | (429) | 24 | (542) | 160 | (3618) |
| Other study staff | 76 | (399) | 0 | (0) | 0 | (0) | 3 | (14) | 0 | (0) | 0 | (0) | 0 | (0) | 0 | (0) | 0 | (0) | 8 | (26) | 0 | (0) | 0 | (0) |
| Data collectors | 402 | (1548) | 936 | (3604) | 6243 | (24036) | 14 | (63) | 144 | (648) | 960 | (4322) | 212 | (488) | 648 | (1490) | 4322 | (9941) | 57 | (177) | 144 | (446) | 960 | (2977) |
| Key informants** | 58 | (0) | 15 | (0) | 15 | (0) | 14 | (0) | 11 | (0) | 73 | (0) | 94 | (0) | 15 | (0) | 100.1 | (0) | 47 | (0) | 18 | (0) | 18 | (0) |
| Drivers | 119 | (560) | 156 | (734) | 1041 | (4897) |  | (0) | 0 | (0) | 0 | (0) | 175 | (4720) | 216 | (5825) | 1441 | (38854) | 30 | (66) | 24 | (53) | 160 | (352) |
| Respondents** | 22 | (0) | 240 | (0) | 1601 | (0) | 8 | (0) | 120 | (0 | 800 | (0) | 24 | (0) | 240 | (0) | 1601 | (0) | 16 | (0) | 120 | (0) | 800 | (0) |
| *Sub(total* | *810* | *(5513)* | *1425* | *(6101)* | *9420* | *(40690)* | *53* | *(393)* | *299* | *(1190)* | *1994* | *(7940)* | *625* | *(7919)* | *1227* | *(9756)* | *8184* | *(65076)* | *177* | *(699)* | *330* | *(1042)* | *2099* | *(6947)* |
| **Data entry/analysis** |  |  |  |  |  |  |  |  |  |  |  |  |  |  |  |  |  |  |  |  |  |  |  |  |
| Study investigators* | 8 | (181) | 82 | (1853) | 400 | (9044) | 1 | (23) | 45 | (1017) | 208 | (4703) | 21 | (475) | 82 | (1853) | 400 | (9044) | 9 | (203) | 45 | (1017) | 208 | (4703) |
| *Sub(total* | *8* | *(181)* | *82* | *(1853)* | 400 | (9044) | *1* | *(23)* | *45* | *(1017)* | 208 | (4703) | *21* | *(475)* | *82* | *(1853)* | 400 | (9044) | *9* | *(203)* | *45* | *(1017)* | 208 | (4703) |
| **Report production** |  |  |  |  |  |  |  |  |  |  |  |  |  |  |  |  |  |  |  |  |  |  |  |  |
| Study investigators* | 8 | (181) | 16 | (362) | 16 | (362) | 4 | (90) | 16 | (362) | 16 | (362) | 8 | (181) | 16 | (362) | 16 | (362) | 10 | (226) | 16 | (362) | 16 | (362) |
| *Sub(total* | *8* | *(181)* | *16* | *(362)* | *16* | *(362)* | *4* | *(90)* | *16* | *(362)* | *16* | *(362)* | *8* | *(181)* | *16* | *(362)* | *16* | *(362)* | *10* | *(226)* | *16* | *(362)* | *16* | *(362)* |
| ***Total time (costs)*** | **1481** | **(11933)** | **2138** | **(13060)** | **12628** | **(63223)** | **168** | **(1065)** | **600** | **(4395)** | **3547** | **(19729)** | **2069** | **(15158)** | **1734** | **(15804)** | **10098** | **(80818)** | **444** | **(2572)** | **674** | **(4401)** | **3695** | **(17368)** |
| See text and Tables 1 and 2 for assumptions for time and cost inputs respectively for a retrospective surveys.  § Population estimation only included if required in study.  * Investigator time inputs are for implementing the informant method, rather than evaluating its validity and feasibility.  ** Costs not attached as they are not normally paid to respondents, participants and key informants. | | | | | | | | | | | | | | | | | | | | | | | | |
